# Experiencing Cancer in Appalachian Kentucky

**DOI:** 10.13023/jah.0203.09

**Published:** 2020-07-19

**Authors:** Melanie McComsey, David Ahern, Robin C. Vanderpool, Timothy W. Mullett, Ming-Yuan Chih, Meghan Johnson, Michele Ellison, Karen Onyeije, Bradford W. Hesse, Eliah Aronoff-Spencer

**Affiliations:** UCSD Design Lab, m.mcomsey@gmail.com; Federal Communications Commission, david.ahern@fcc.gov; NIH, vanderpoolrc@mail.nih.gov; University of Kentucky, timothy.mullett@uky.edu; University of Kentucky, mch266@uky.edu

**Keywords:** Appalachia, cancer, broadband, communications, connectivity, rural health

## Abstract

**Figure:**
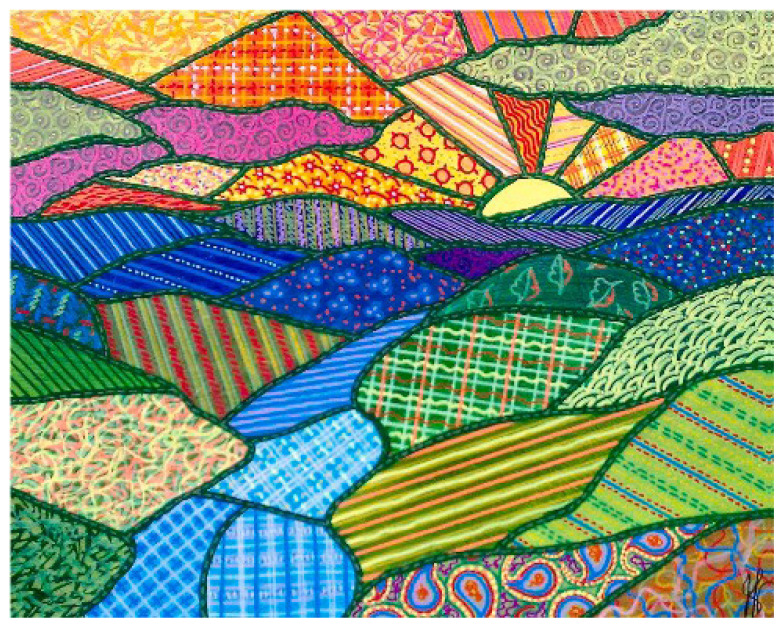
Quilted Appalachian Sunset ©2011 Jim Harris jim-harris.pixels.com

Quilted Appalachian Sunset

©2011 Jim Harris

jim-harris.pixels.com

Nothing tells the story of people working together better than a community quilt. A diversity of talents, colors, and materials brought together through skill and shared purpose. Perhaps never before have we as Americans needed a stronger reminder that many hands make short work of big problems. The work presented here by the L.A.U.N.C.H. Collaborative offers a new framework for health care that could be compared to a digital quilt, powered by community-based participatory design, with lived expertise and the newest advances in broadband-enabled connected health solutions. This work demonstrates the value and need to engage local communities and what can be learned when beneficiaries and traditional caregivers work together to develop healthcare solutions.

## INTRODUCTION

In Appalachian Kentucky, cancer incidence and mortality are higher than in other parts of the country.^1^ Patients diagnosed with cancer in rural Appalachia often face additional challenges to managing their symptoms and receiving care, including economic insecurity, geographic isolation, transportation challenges, comorbidities, and limited access to specialty care.^2^ Meanwhile, lower rates of broadband availability and Internet adoption relative to other parts of the country^3^ limit the reach and success of connected health solutions that may have the potential to address these challenges. In Appalachian Kentucky, as in many other rural or minority communities in the United States, elevated cancer incidence and mortality is a problem of access to existing interventions and care; indeed, the American Cancer Society has estimated that nearly one quarter of U.S. cancer deaths could be averted if all Americans had access to the highest quality care.^4^ In this sense, then, improving outcomes and quality of life for many patients is a problem not of innovation of new therapies or complex new technologies, but of innovation of processes of healthcare distribution and implementation.

The research presented here was conducted as part of a public–private collaborative called L.A.U.N.C.H., Linking & Amplifying User-Centered Networks through Connected Health, which addresses challenges at the intersection of healthcare and connectivity.^5^ The project seeks to bridge community-driven design, broadband connectivity, and connected health technology to improve cancer outcomes and quality of life for patients in rural and historically underserved areas. Indeed, the President’s Cancer Panel Report specifically recommends the expansion of Internet services so that the full benefits of connected health can be achieved. Cancer—with its complex biology, multispecialty care teams, transitions between treatment phases, experience of symptoms related to treatment, and profound impact on the lives of patients and families—is an area of healthcare likely to benefit from improved coordination, communication, information access, and health behavior change facilitated by connected health solutions.^6^

This kind of process innovation, in complex systems involving humans and technology, is never exclusively about functionality, but also about meaning. Anthropologist and physician Arthur Kleinman^7^ distinguishes between “disease,” which is a biomedical construct, and “illness,” which characterizes the “innately human experience of symptoms and suffering.” He argues for an approach to clinical care that puts this experience and meaning of illness at the center; that includes “empathic witnessing of the existential experience of suffering” as well as “practical coping with the major psychosocial crises.” This project’s approach assumes that, in order to be successful, healthcare innovations should be meaningful, intelligible, empathic, and fit with local ideas of being a good and healthy person.

In this spirit, the L.A.U.N.C.H. project’s contextual inquiry took an ethnographic approach. The team sought to collaborate directly with local stakeholders, including cancer patients, caregivers, and healthcare professionals, in order to understand their experiences with illness and caring for the ill. Ethnography is a methodologic perspective, typically associated with the discipline of anthropology, that seeks cultural meaning in empirical data about human behavior. Ethnography is thus both observational and interpretive. Anthropologist Clifford Geertz^8^ has likened the ethnographic method to the method of clinical inference. “The diagnostician,” he writes, “doesn’t predict measles; he decides that someone has them” based on a set of signifiers (symptoms). Similarly, ethnographic data start with observed symbolic acts and seek to place them in an intelligible frame—to make sense of them. In the analysis that follows, we make sense of our data by interpreting them within a set of cultural frameworks, which, like a diagnosis in the context of comorbidities, lend coherence to the complexity of observable signs without suggesting that all signs need fit in the frame.

Another important feature of the ethnographic method is that its insights are delivered from the perspective of the participants, to the extent possible. Throughout this paper, generalizations about “Appalachians” are made on the basis of how participants perceived themselves as “Appalachian,” rather than on the basis of any systematic comparison with other populations. Importantly, the reader will note that participants mostly characterized themselves and their communities as “Appalachian” not to distinguish themselves from other Americans, but in contrast to the institutional cultures—the healthcare system, the dominant economic system, and information-technology systems—that play a role in the cancer experience. Thus, the designation “Appalachian,” or other designations setting our interviewees apart culturally, should be taken to designate contexts in which participants saw themselves and their communities as distinguishable relative to specific institutions.

Thus, the findings presented here may be relevant for understanding some aspects of the cancer experience for other American subpopulations to the extent that people in other subpopulations also struggle to interface with the institutions that exist, supposedly, to help care for them. These common struggles between institutions and communities may productively be understood within the framework of a “development encounter.” Anthropologist Arturo Escobar^9^ defines “development” as a Western cultural narrative, predominant since the end of World War II, that proposes to replicate the conditions of the most “advanced” societies the world over: “high levels of industrialization and urbanization, technicalization of agriculture, rapid growth of material production and living standards, and the widespread adoption of modern education and cultural values.” The development narrative is put into practice by institutions, such as governments, universities, healthcare systems, telecommunications companies, and others, which seek to bring progress and prosperity to “underdeveloped” places and people.

Such development encounters between would-be developers and the “underdeveloped” share common features wherever they have taken place. In *Uneven Ground: Appalachia Since 1945*, Ronald D. Eller^10^ traces the history of the failure of public development strategies in Appalachia after World War II. Starting the 1960s with several federal initiatives aimed specifically at Appalachian economic development, the region came to be constructed by politicians and media imagery as “chronically underdeveloped” and as a symbol of the failure of the American promise of prosperity, argues Eller. He adds that despite the special attention the region has received and the idealism of many antipoverty initiatives, material conditions in Appalachia have, if anything, become more unequal relative to other parts of the U.S. in the present day. If this failed promise of public development rings true for other American subpopulations, it is because, according to Eller, the “uneven ground of Appalachia” is not some other America: it is America.

This paper models an alternative approach to addressing disparities in healthcare and connectivity that goes beyond the typical development encounter paradigm. We begin with an ethnographic account of how eastern Kentuckians experience illness, drawing on first-hand testimony from local cancer patients, their families, and their healthcare providers. A discussion of the cultural frameworks and social facts shaping the cancer experience gives insight into how connected health solutions may be co-created and implemented in collaboration with local stakeholders. We argue for an approach to innovation that is locally meaningful, community-driven, and that can build lasting coalitions among institutions and people, in order to ensure equitable access to health and connectivity resources.

## METHODS

The primary data for this study come from ethnographic interviews conducted with cancer patients, cancer survivors, and caregivers from eastern Kentucky. Eleven interviewees participated in ten ethnographic interviews (Table 1), including three men and eight women. Four were cancer survivors, three were current patients, and four had cared for someone with cancer. Participants came from nine different counties in eastern Kentucky and had been affected by a variety of different cancers. Participants were primarily recruited onsite at healthcare facilities and interviewed either on the spot or at a later date. This sample size and sampling method are typical of ethnographic studies. Some additional qualitative data came from informal conversations with “community partners” in Kentucky, who were contacted and engaged through networking and respondent-driven sampling. Community partners consisted of 51 individuals in sectors across healthcare, telecommunications, government, nonprofits, and research. Where applicable, this information is relied on to supply contextual information or to corroborate information from the interviews. All names used in this document are pseudonyms, and some specific place names and institutions have been changed to protect the privacy of participants.

Ethnographic interviewing was conducted by two different interviewers in the following eastern Kentucky counties: Clark, Jackson, Johnson, Knox, and Perry. Some recruiting and interviewing took place at the University of Kentucky Markey Cancer Center (MCC) in Lexington, and at Hazard Appalachian Regional Healthcare (ARH) Regional Medical Center in Hazard (Perry County). Some interviews were conducted in patients’ or survivors’ homes, workplaces, or other community-based locations.

Individual ethnographic interviews were conducted either with one individual participant or with family members or friends involved in caring for the patient. Interviews lasted approximately 60 minutes and were audio-recorded. The interviews resulted in over 12 hours of recordings and a corpus of over 97,000 words. The interview data were coded using qualitative data analysis software and following a bottom-up strategy in which coding categories were identified as they emerged in the data. The purpose of these interviews with cancer patients and their caregivers was to understand their personal experience with cancer; understand their social context within their community; understand aspects of their values or worldview that may be relevant to successfully implementing connected health interventions; understand the ecosystem of resources available to them; and understand gaps in those resources.

### CULTURAL FRAMEWORKS

How patients and caregivers in Appalachian Kentucky experience cancer may be understood through the lens of several related and complementary cultural frameworks. Cultural frameworks are interpretive schemas that help organize and make sense of cultural information, including values and cultural narratives. Frameworks, values, and narratives are not usually consciously articulated by people but can be discerned through a close analysis of what people say, how they say it, and how they behave. They are the “common sense” that is taken for granted and undergirds how people think and how they act. In this sense, frameworks, values, and narratives are often taken as factual within a community and used to guide appropriate behavior. However, because communities are not monolithic and human behavior is not generally rational, frameworks, values, and narratives within a given community may be contradictory, contested, or in tension.

Appalachian Kentuckians’ cancer experience is also shaped by several social facts. For the purposes of this report and departing slightly from Émile Durkheim,^11^ the social scientist with whom the term is most often associated, a social fact is a social institution or structure that shapes how people think, act, and interact. Examples include the norms surrounding institutions like religion, family, community, and marriage. One way that social facts differ from cultural frameworks is that people are often able to consciously describe social facts and some of the ways social facts affect their behavior. Social facts may be more stable and more resistant to change than cultural frameworks, values, and narratives. They are less likely to be in contradiction within cultures and are often invoked to distinguish “different” cultures.

**Figure 1:**
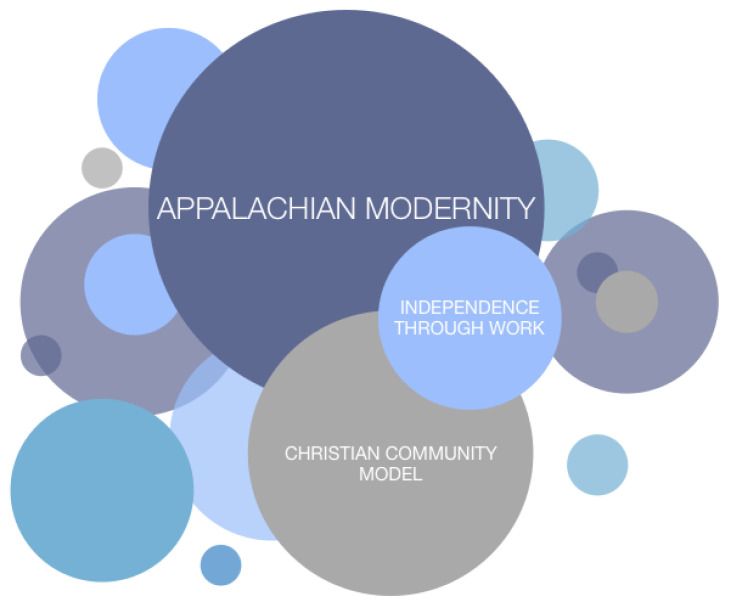
Model of the Interrelated Frameworks

Three primary cultural frameworks were developed to understand how patients and caregivers in Appalachian Kentucky experience cancer. The first, we identify based on themes in our own data, and call the Christian Community Model. The second, the Independence-Through-Work Model, has been described in previous ethnographic work.^12^ And the third, the Appalachian Modern Worldview, takes inspiration from the extensive sociological literature on “alternative modernities” (Figure 1). We developed these cultural frameworks by analyzing the values and cultural narratives voiced by participants during ethnographic interviews and other interactions with researchers, and then identifying patterns in these signs. It should be noted that there are always many cultural frameworks one could invoke to interpret an instance of human behavior; the three discussed here were the ones we deemed most useful to understanding the illness experience of our participants.

A given individual’s worldview may be understood relative to multiple, interrelated cultural frameworks, cultural narratives, and values. In eastern Kentucky, the Independence-Through-Work Model, the Christian Community Model, and the Appalachian Modern Worldview are shared by many. Other cultural frameworks, represented in this figure as unlabeled circles, are also at play, and may be shared regionally, or not.

The cultural frameworks outlined in this paper may be used to understand not just how Appalachians experience cancer, but also how they experience many other facets of life. Because cultural insights are often transferable across domains, there is an opportunity to leverage these insights not only in co-creating tools for connected cancer care, but also for imagining and implementing other innovations, in healthcare and beyond. For example, Appalachian frameworks for understanding flows of information and power, as well as progress and development, are directly relevant to the domain of broadband access and adoption, telehealth use for COVID-19 and other diseases, and other initiatives aimed at improving wellness and wellbeing.

#### The Independence-Through-Work Model

Independence-Through-Work is a key component of the Appalachian Modern Worldview, described below. In this model, independence, personal virtue, and individual success are deeply interwoven with ideas of work and providing for family. Independence does not mean individual liberation or freedom from attachments; instead, it means having the means to establish oneself as an adult who is economically and socially secure. Like other aspects of the Appalachian Modern Worldview, Independence-Through-Work is seen as a value system under threat. Dependence on the government and other kinds of “charity” are associated with negative “hillbilly” stereotypes about poverty, ignorance, and laziness. Other ethnographic work in Appalachia has shown that Appalachians are also resistant to political and economic policies seen as creating a system of dependence rather than promoting independence.^12^

#### The Christian Community Model

The Christian Community Model complements the Independence-Through-Work Model by embedding the economically independent family unit in a system of broader social relations. A shared Christian worldview is taken for granted among Appalachians and provides a common vernacular for forming and maintaining relationships. The Christian Community Model posits a set of social obligations to be fulfilled according to specific social categories. These may take the form of practical help such as raising money or praying for someone. But these also take the form of a personal responsibility to behave, or even feel, a certain way. The Christian Community measures and sanctions moral behavior.

#### The Appalachian Modern Worldview

Modernity is a set of material conditions, a form of social organization, and a worldview that arose during the Enlightenment in Europe and has since spread around the globe. Readers will be familiar with the most common modern material conditions, such as the market economy, urbanization, the rise of the nation state, the rise of representative democracy, secularization, and the use of science as a method for creating knowledge. Along with these material conditions, a modern way of understanding the world, or a worldview, also began to emerge and spread in the 17^th^ and 18^th^ centuries. The modern worldview takes for granted that progress is good and attainable through work; that individuals have inalienable rights; that reality can be analyzed in discrete categories; and that religion is just another ideology among many. The emergence of this type of “classic” Modernity in Europe has been extensively described in the social sciences,^13^ as has the notion that as Modernity spread beyond the West, it was not necessarily taken up wholesale, but “hybridized” with other systems and worldviews. These modern societies beyond the West have been variously referred to as “hybrid,” “alternative,” or “multiple.”^14^ Indeed, any notion of a “classic” modern worldview must be considered a theoretical construct, as most individuals’ worldview will borrow from this and other sets of values. A small body of literature has examined the intersection of Modernity and traditional Appalachian culture.^15^–^18^ Here, we posit one alternative Modernity, an Appalachian one, as it helps make sense of the worldview of the people we interviewed. The Appalachian Modern Worldview as described here is based only on the empirical evidence we collected, rather than on any preexisting theoretical model.

The Appalachian Modern Worldview melds aspects of a classic modern worldview with local traditions, resulting in a unique set of values. Appalachians recognize that their values and way of life differ from those undergirding modern institutions. This is a source of both pride and cultural insecurity—the latter due to a long history of negative stereotyping of “hillbillies.”^19^ Appalachian Modern values may include, for example, frugality and self-sufficiency, in contrast to the modern values of consumption and materialism. Appalachian Moderns may value familism and regionalism over the individualism of Modernity; they may value enjoyment of experience over rational efficiency; they may value spiritual experience and emotional sensibility as ways of knowing, in addition to or instead of scientific knowledge.^17^ These are just a few examples of how an Appalachian Modern worldview can differ from a classically modern worldview.

### EXPERIENCING CANCER

The three cultural frameworks described above help make sense of how Appalachians experience many realms of life, both in the context of cancer and beyond the context of cancer. Our interviewees talked about diverse facets of the cancer experience, from their experience of pain and suffering to their expectations about the role of the government in providing health insurance. The analysis that follows is organized according to the most prominent of these themes:

- The experience of suffering and distress
- The roles and responsibilities of community, family, and the individual
- The role of prayer and faith in healing and social life
- Economic models affecting financial situations
- How to acquire, use, and make sense of knowledge and information
- The experience of communicating in diverse settings with diverse people
- How local values intersect with institutional notions of progress

In the sections that follow, each of these themes will be discussed, showing how they cohere in the three important cultural frameworks, and how they are illustrated with the voices and words of 11 cancer patients, survivors, and caregivers from Appalachian Kentucky.

### SUFFERING AND DISTRESS

“Distress” emerged naturally as a theme that interviewees reflected on in recounting their cancer stories, but it was also one of the main areas of interest for this research from the inception of the project. This is due to the fact that monitoring symptoms and distress of cancer patients can have a significant impact on cost of care, quality of life, treatment outcomes, and survival.^20^,^21^ In the context of this scientific narrative, “distress” is a type of data, the collection of which allows the application of appropriate treatments and improves outcomes for patients. Indeed, Commission on Cancer (CoC) accredited cancer centers must meet Standard 3.2: “Cancer programs must develop a process to incorporate the screening of distress into the standard care of oncology patients.”^22^ While the Standard outlines specific requirements for distress screening, specific tools and methods are not mandated.

Participants in the current study did not use the word “distress” or talk about clinical distress monitoring. But they did speak about the physical pain and emotional suffering associated with cancer and pointed to ways they coped with this suffering.

#### Individual Suffering Versus Social Suffering

When interviewees spoke about their own cancer-related distress, they rarely, if ever, adopted the idea of it being treatable by a medical professional. Instead, distress, or suffering, were experienced as part of ongoing, even lifelong, moral and social practice shaped by the Christian Community Model. In the model, excessive individual suffering is taboo because it can cause distress for loved ones. Therefore, talk about distress was often bracketed by expressions of positivity or couched in delicate euphemism. However, speakers did plainly acknowledge any distress caused by another’s distress. This kind of “social suffering” is an important component of the Christian Community Model because it indexes the strength and value of social bonds. As a result, some participants described social suffering as a form of comfort.

The stories in this section illustrate the meaning of suffering in Appalachia, how Appalachians experience suffering, and their approaches to alleviating suffering.

#### Physical Suffering

Even in the context of a cancer-focused interview, talk about personal physical distress was rare. Indeed, two interviewees, a survivor and a current patient, recalled feeling mostly good after treatments, and, even when prompted, offered no information about physical distress. In her interview, Laura emphasized that the speed of her cancer journey kept her from feeling much at all, and her memory of treatment centered on the “smiley faces” in her journal:

Most of the time [I felt good after treatment], yeah, because I had even kept a little pocket calendar and I didn’t document a lot of it as far as in writing as far as each day. But I did make a point of every day on that calendar, I would either make a smiley face, just a straight face, or sad face. That indicated how I felt. Looking back on that calendar, there’s way more smiley faces than there are anything else (Laura 09:48).

Bobby, although in the hospital for an unrelated health issue at the time of his interview, maintained a nonchalant attitude about his treatments for pancreatic cancer:

Chemo. Tolerated that real well. I don’t know if I ever had any nausea or anything. I did have hair loss, but I had my energy and stuff, I had my appetite and everything….Physical activity, worked, and stuff, and everything (Bobby 00:15:34).

When participants did discuss physical distress, they tended to bracket it with expressions of positivity. In addition to her cancer, Penny has had several serious injuries, including a broken back 10 years ago, and the fall she described in the excerpt below, which resulted in another back injury. After the description of the injuries and the acknowledgement that they cause her pain, she emphasized that on “good days” she doesn’t feel like she has cancer at all. Remarkably, she even claimed not to know whether cancer causes pain.

Then a year ago, I fell down eighteen steps at my daughter’s home. I broke six vertebrae, five ribs, my sternum and my hand, a bone in my arm and one in my foot. So I had to go off chemo for about eight weeks and the cancer really ate at those eight weeks…I am on Morphine twice a day to help with it and to help with, I guess the pain from the cancer. I don’t know if there is pain that comes with cancer or not. It takes care of it… Then there are good days when I feel like if someone didn’t tell me I had cancer I wouldn’t know I did (Penny 10:34).

#### Social Suffering

Descriptions of physical suffering were not limited to patients; caregivers also described the physical toll of caring for someone with cancer. However, because of the social nature of this suffering—suffering on behalf of another—caregivers also expressed the joy that comes with work in the service of a loved one. Wanda claimed to “love” taking care of her sick husband, despite how tiring it was. Indeed, she even presented her weight loss—a possible sign of distress in a medical framework—as a silver lining.

Oh yeah, he was incapacitated the whole time he had cancer. I was taking care of him. It was just, very, very tiring. It was—but I loved it. I took care of him. I was not bitter for it. I would do it again. Actually, I lost 25 pounds in 20 months (Wanda 00:25:37).

Wanda probably genuinely experienced this joy in social suffering; but her words were also a description of ideal, expected behavior, given a Christian Community worldview. Like the physical distress of a cancer patient, her tiredness is taken for granted and naturalized. But unlike the physical distress of a cancer patient, her tiredness is an index of love for her husband.

#### Social Bonds As a Relief from Suffering

According to the interviewees, social bonds are not only indexed and strengthened through suffering, they can also serve as a comfort from suffering. Wayne recounted how receiving cards in the mail helped him through physical pain he described in the most extreme terms:

There were lots of days that you would have really thought you would’ve had to have died to have felt any better at all. But what I didn’t realize would happen, is even on those days when I would take one step forward and three steps back, I would go to the mailbox and there would be a card from the last person I would have ever thought I would have heard from, encouraging me. So, you would have things like that that would help me through that day in through the next (Wayne 00:09:57).

Unmitigated descriptions of physical distress appeared only when caregivers discussed the physical distress of their patients. Caregivers tended to follow these descriptions of physical distress with a coda linking that distress to their own emotional suffering. In effect, they validated the taboo against patients talking about their own physical pain by illustrating the distress this pain caused them as caregivers. Jane, the mother of a 20-year-old with rhabdomyo-sarcoma, described her son’s physical suffering with passion and indignation. In the excerpt below, she positioned herself as suffering alongside him to the best of her ability.

My 20-year-old son uses a walker, and sometimes his legs just stop, and he wobbles. He has had so many side effects. He just spent a week in the hospital last week with [an infection]. I spent a lot of time sleeping on couches in that hospital (Jane 00:03:50).

Claire waxed sentimental about her father’s eye color, recalling his final days in the hospital. Her description of his pneumonia caused by an undetected yeast infection is one of few instances in which an interviewee mentioned unreported symptoms contributing to major medical complications.

The last medicine they had him on, the last chemo medicine, it had caused yeast in his throat. We didn’t know. Where he was trying to eat, he was aspirating some of it. So, it caused pneumonia. That’s how he ended up in the hospital the last time…His eye color changed. He had hazel eyes. His eye color went blue. I guess from the radiation. It was, I just kept looking at his eyes, you know. ‘Cause I’d see dad’s face and see mom’s eyes (Claire 38:50).

#### Emotional Suffering

Talk about emotional suffering was much more common than talk about physical suffering. This is probably because emotional suffering is more likely to be moral or social in nature than the individualized suffering of physical pain, thus making it more culturally appropriate in the context of eastern Kentucky. The language interviewees used to talk about emotional distress included a range of typical descriptive terms, as well as colorful euphemisms. Talk about emotional distress, like talk about physical stress, was often bracketed with expressions of positivity which had the effect of minimizing the self-indulgence of the speaker.

An excerpt from Bobby exemplified several of these key patterns. He began and ended this short narrative by answering positively to the interviewer’s inquiry about his health. Sandwiched between were descriptions of his emotional distress, ranging from a direct expression of anger to the euphemistic “I don’t feel normal,” to the metaphorical, “This cloud’s a-hanging over me.” He also appealed to his moral obligation to keep a positive attitude: if God is working through the doctors, he is obligated to place his faith with them. To be angry is to lack faith.

So, I guess, I’m doing good. I have my moments where I get angry and I say, ‘Again, all this?’ I say, ‘Well, but Bobby, you beat it last year.’ And I know I overcome it last year, and I know, and when you got doctors and nurses and whoever who says, ‘Well, God works through us.’ That’s who you want on your medical team. And I feel like, well, if He did last year, He’ll do it again this year. But some days you’re thinking well, this cloud’s a-hanging over me again and I don’t feel normal. Then some days I feel normal (Bobby 00:09:32).

#### Reciprocal Social Suffering

Overwhelmingly, patients and survivors said that the hardest part of their illness was seeing the suffering it caused their loved ones. Interviewees talked openly about this kind of reciprocal social suffering. In the Christian Community Model, not only is this kind of suffering socially acceptable to experience but talking about it reinforces the social bonds themselves.

Both Mary and Hazel contrasted their own stoicism, even in the face of death, with the “devastation” and sadness their family members felt. Mary’s observation that when one person has cancer, “it is a cancer that the whole family gets” put words to an important theme that recurred throughout the interviews: the blurry line between self and family. This theme is discussed in more detail, below. Note that both speakers ended their narratives with a coda that framed the reciprocal social suffering as something positive—because it is an index of love.

If I had to say the hardest thing about everything as far as the emotions was the look on my husband and my children’s face. Because it was, that’s one of the things that’s the hardest. I could handle the situation and I could handle the death part of things, but it was the look on their faces, you know the devastation for them that I worried about. When you have the cancer, a disease like cancer, it is a cancer that the whole family gets. It’s not just the patient because everybody is dealing with it. So, I’ve just been so fortunate and so blessed (Mary 01:03:26).

And so I went in [for surgery] and I wasn’t afraid. I didn’t try to run away, I didn’t cry, the only thing that worried me… [crying]...sorry. I was worried about my daughter because she was so sad. And it just, I couldn’t stand the thought of her being sad...So that was what bothered me more than anything, but as far as being afraid, I feel like God was with me and that He was telling me either way, you’re gonna be fine because if you know, you die you go to heaven, if you don’t then then you’ve got your family. So, either way it’s gonna be okay (Hazel 00:17:18).

#### Talking About Emotional Suffering

Despite interviewees suggesting the social acceptability of social suffering, they used a range of euphemisms, colloquialisms, and nonclinical terminology to talk about their emotional distress. These euphemisms likely served to minimize the severity of the distress while also capturing some nuance lacking in clinical terminology.

And when I realized near the treatments, with **all the nerves and things I was having**, I couldn’t have done it by myself. So you know, I can see how patients can OD [overdose on pain medications] and you know, then bad things happen (Wayne 00:09:07).

My sister was battling cancer too, at this time, my real close sister that lived [out of state]. She was battling cancer, so I was **all freaked out** over her already. Then now my husband got diagnosed (Wanda 00:20:29).

All he did was just sit. **He couldn’t motivate and get around**. If he could’ve just walked during all this, it would’ve been so much better for him mentally (Wanda 00:37:18).

You **go through the grieving process**, not only for your health, but when you lose your career and everything (Mary 00:25:48).

It’s **very scary**, just going into that unknown (Laura 08:21).

**It’s terrifying**. There’s times when I have to tell him, ‘I’m going to go get you an ice pack.’ Just so that I can walk out of the room and try to recompose myself (Jane 00:34:00).

**I felt lonely** because I didn’t have any hair or nails (Laura 34:33).

In general, participants did not use clinical terms such as “anxiety” or “depression.” Where those terms were used, it was often to describe an explicit stance against their validity. In the following excerpt, Hazel questioned the medicalization of sadness and admitted she had not told her providers about feeling sad.

I don’t even tell ‘em about depression ‘cause I should be able to fix that, but I mean, that’s a brain thing, I shouldn’t have to have medicine to make me happy for heaven’s sakes, I shouldn’t be sad. I mean, for now I’m healthy and I’m getting to see my grand-babies and I get to see my daughter, and I’m blessed….But no, they [healthcare providers] don’t talk to me, I guess they would if you told ‘em or something, I never told ‘em (Hazel 01:03:11).

Hazel brought home the key problem for clinical models of emotional distress in the context of eastern Kentucky. She saw her depression as not medically treatable because within the Christian Community Model, it should not exist at all. Her strong relationship with her family “should” be enough comfort in the face of suffering.

The stories in this section have illustrated the different values ascribed to personal versus social suffering in Appalachia. Many Appalachians seem to experience suffering in the context of social relationships and obligations. Relatedly, approaches to alleviating suffering—although not always successful—emphasize an appreciation for social connections.

### COMMUNITY, FAMILY, AND THE INDIVIDUAL

Sick people need help, and this was a key theme for the participants we talked to. When it comes to help, the Independence-Through-Work Model and the Christian Community Model can be in tension: while the Christian Community Model suggests that helping others is good, the Independence-Through-Work Model suggests that receiving from others is shameful.

One of the ways Appalachians resolve these competing values is by blurring the line between self and family. Recall, for example, Mary’s observation, “When you have the cancer… it is a cancer that the whole family gets” (01:03:26). In Appalachia, one’s family is considered an extension of one’s self, so that “help” from a close family member is not considered freeloading. Wanda’s husband, for example, who had lung cancer, would not accept caregivers from outside his immediate family.

He didn’t want me to have the babysitter come and sit with him. He didn’t want that. It sort of made me having to stay with him 24/7, which I did. He would let maybe my sister-in-law sit with him for a little while, while I had to run an errand or something (Wanda 00:38:38).

But many participants extended the “family” metaphor beyond their blood relations, thereby expanding the realm of possible helpers. Several stories follow below of “church families” and “work families” rallying around a person in need.

Another way Appalachians resolve these competing values is by reframing excessive independence as a lack of humility—a negative value within the Christian Community Model. As breast cancer survivor Mary explained, a pastor she met at her radiation treatments taught her that allowing herself to be helped was a form of Christian generosity itself. Mary hoped to pass this wisdom along to others facing cancer.

The stories in this section illustrate the ways in which Appalachians help each other in culturally appropriate ways, and the types of help that were most valued by participants.

Box 1 **Words of Wisdom**

There are just so many things that you know as hindsight, you look at and one of the things, this little pastor—we were the first two to get our radiation treatment every morning. I told him that I’d always been the person that did things for people and I’m on the receiving end and it’s real hard for me to allow people to help me. He told me, he gave me a suggestion. He said, ‘Do not deprive people of the blessing of helping you because they can’t do, you know sometimes they don’t know what to say. They don’t know what you need. But they want to do what they can. Whether it’s coming in and doing your dishes and mopping your floor. Let people have that blessing because it’s more of a blessing to them than it is to you.’ So, he said just let them do what they can. That was one thing, I had to learn to be humbled. I had to be humbled, basically, to see how important it was for people to be able to reach out and to help you. So, I would say let them have their blessing and let them come and do what they can for you. Let them give you money if they want to give you money to help and bring you meals if they want to do that (Mary 01:07:04).

#### How to Help in Appalachia

In Appalachia, practical forms of help are considered more than transactional— they are meaningful expressions of caring. Bobby concisely illustrated this cultural narrative when he claimed that people interpret his nonchalant acts of goodwill as “caring and giving.”

Well, that’s like me, if I know where there’s a need, I go ahead and do it. I’ve told people and I’ve said, I don’t do it for compensation or recognition. I said, it’s just something that needs to be done. People told me, they said, ‘Bobby, because you’re always caring and giving to other people, that’s probably why so many good things is coming back to you.’ I say, well, I guess it’s like that old saying. How you treat people comes back to you (Bobby 00:37:02).

Although Bobby—and others—stressed a lack of expectation of compensation for their goodwill, he acknowledged that “how you treat people comes back to you.” This is an example of one aspect of the Christian Community Model, in which caring relationships are sustained over time through material social obligations.

#### Help from Family Members

Patients and survivors described with some reverence the practical help they received from close family members. Much of this help was intimate, taking place in private spaces and involving private rituals. The intimacy of caring for a sick person or being cared for is one example of how the need for help blurs the line between self and other. Hazel described her sister’s dedication to helping her at every moment. An anecdote about a prank her sister played, by videotaping her while she was medicated and praying, made a lighthearted comment on the invasion of privacy that comes with being sick.

I think what touched me more than anything with [my sister] was that she stayed up…She missed work at all three jobs the whole time I was in the hospital. And never left my side. She slept in there in a little hard couch, when I’d moan or groan, she’d get up and see what was wrong with me. Now, she did videotape me, which was very bad of her. They give me all the pain medicine they could, and she videotaped me praying (Hazel 00:36:13).

Several patients mentioned practical help they received consisting of records-keeping and the management of information. In these examples, the line between self and other is further blurred as caregivers served as the very memory and voice of the patients they cared for. These examples also show how the skillsets of family members may be recruited in the service of a patient and become a form of capital that can be leveraged in institutional settings.

And my wife is an engineer by vocation. And she had just been made project manager over a [large] project. So, for her to be there with me, day in and day out, just was not feasible. But one of the things she did, she managed my healthcare like a project… Medications, every medication I took was documented. If I had any kind of side effects, it was documented (Wayne 00:33:44).

And my son-in-law is a pathologist, so he keeps up with the tumor board councils…Very fine, and my daughter that passed away was a cytologist and histotech[nician]. She knew of all the cancer, the kind that I had, and the prognosis. She would tell me about my treatment that I would be taking. Not that I just miss her from being gone from my daughter, I miss her as the information I needed (Penny 07:25).

#### Help from Church Families and Work Families

Interviewees had also received practical help from their “church families” and “work families.” As Claire explained, when your church family helps you, it is an “offering” that is acceptable to take. “Offerings” are contrasted with “charity,” which is not acceptable. According to Claire, the distinction between an “offering” and “charity” depends on who is giving it and the meaning behind it. Thus, invoking the metaphor of “family” for groups beyond immediate relatives allowed for an expanded circle of helpers considered socially acceptable in the local worldview.

I know a lot of people are too proud to ask. Because they feel it’s charity...But if it’s from your family or a true friend or from your church family, believe that it’s a true offering and it’s not charity. They really mean it. Take them up on it (Claire 49:24).

Mary described some of the practical help she received from her church family:

Now for the chemo, usually a person from church would take me because you have to sit there for three hours and [my husband] just couldn’t take off work all the time for that. I was very fortunate to have a really good church family to help through all this and they brought meals to the house and things. But it was, I was very thankful for it (Mary 00:24:49).

Some participants extended the family metaphor to their places of work.

Mary described the fundraiser held by her colleagues:

[My husband] had taken off so much work for my surgeries and everything, so we just I was just very fortunate to have my church family and my, actually my [workplace] family, did a fundraiser, and raised the money for me to buy my anti-nausea medicine. Because my insurance, even though I had insurance, I was still—I forget it was like $300 a prescription every time I had to go for treatment (Mary 00:47:59).

Although Jane didn’t specifically use the word “family,” she talked about the “love” and regular communication shared by her son Jake and his work colleagues in contextualizing the fundraiser they held for him.

But everybody loves him [at work]. They put Jake off on a leave. They’ve put him off for a year, but his job was waiting for him. The whole team still texts him. We walked in there and the manager just...He loves Jake and he comes up, ‘We’re praying for you buddy.’ Two weeks ago, they had a cookout. They brought the big Pepsi truck out and they had a big cookout and they raised everything that was sold there that day, went for Jake for the weeks of radiation so that I could be with him (Jane 00:16:34).

The metaphor of “family” was invoked, then, to describe socially acceptable ways of giving and receiving help in Appalachia.

#### Reciprocity

Another socially acceptable way to receive help in Appalachia is in the context of reciprocal giving. Bobby explained that he accepted help from a neighbor whom he had helped in the past. But Bobby was also quick to emphasize that the decision to accept help should be left to the individual, and that help should not be forced upon anyone. Although Bobby acknowledged receiving help when he was sick, he stressed that this was out of necessity and not out of a lack of appreciation for individual autonomy and independence.

So, people should make that decision on their own when they feel like they need help and stuff. Sometimes we have to put our pride or whatever to the side. Now, I did when I was in the hospital this year, the last week of June until I got out 4th of July. Now, [my neighbor] came and done and mowed the yard and fed the cats. I said, so, I liked that yard a certain way. I live in the homeplace, I’m a widower. I said, but I like everything a certain way. So, he said, ‘Bobby, I’ll take care of it.’ He said, ‘You’ve always helped me, brought me pies and cakes and stuff.’ He said, ‘Like I don’t need them.’ He said, ‘You see that,’ he said, ‘You’ve always asked about me when I’ve had something with my back or something.’ Just as far as the individual, I think they’ll know when they need help and leave that up to them. You can encourage somebody, but I don’t think you should force somebody (Bobby 00:40:51).

The stories in this section illustrate how Appalachians help each other in culturally appropriate ways by appealing to notions of family, reciprocity, and by respecting the autonomy of the individual. Overall, participants valued all kinds of help, from the intimate to the quotidian to the financial.

### HEALING, PRAYER, AND FAITH

Interviewees spoke extensively, without prompting, about the role of faith and religion in their cancer journeys, making these themes impossible to ignore. Even though it is taken for granted in eastern Kentucky that almost everyone is a Christian, there are a plethora of Christian faith traditions in the region.

Although often described as “homogeneous” in terms of race and ethnicity, Appalachians see themselves as diverse, partially because of the diversity of faith traditions. One of the most iconic Appalachian faith traditions is the Holiness tradition, a Pentecostal tradition sometimes stereotyped as “snake handlers.” Interviewees noted that even town-dwelling Appalachians sometimes stereotype the Holiness as backward. Holiness churches are apparently associated with rurality, while folks in the county seats are more likely to identify with Baptist, Methodist, or other more mainstream faith traditions.

One significant difference between Holiness and Baptist traditions is their beliefs about healing. A belief in “miraculous healing” is a hallmark of the Holiness tradition. In miraculous healing, the “natural” course of events is altered by an intervention from God via the Holy Spirit. According to the Great Commission (Matthew 28:16–20), a key tenet of Holiness theology, true believers will be known by their ability to heal the sick by laying hands on them. The laying on of hands is practiced in many Holiness churches in eastern Kentucky. Of our interviewees, only Penny expressed an overt belief in miraculous healing:

I have a very fine church family. My cousins are uplifting to me and the neighbors I have are believers in the healing through Christ that can be given, and I do too. I really believe it can be stopped or it can be completely healed away. It’s just at His choosing (Penny 08:46).

The only other reference to miraculous healing by a participant occurred in a story Bobby told about a pastor he saw on television. With characteristic insight, Bobby pointed out that a belief in miraculous healing is often stigmatized among medical professionals—who, in Kentucky, may otherwise openly express religious inclinations—and that this stigmatization runs counter to regional beliefs.

[The pastor on TV] spoke about his mother-in-law that had breast cancer and maybe a brain aneurysm. He said she was miraculously healed. I thought well, I guess you have to put faith and believe in that. Some medical people don’t want to bring that up and talk about it. But now, I don’t know about anywhere else...Faith and religion is deeply ingrained in this region (Bobby 00:20:46).

In contrast to miraculous healing, providential healing is when God acts through earthly humans and events. In faith traditions that subscribe to a notion of providential healing, God’s love is seen as a creative power that manifests on earth, often through actions. Bobby’s comment, cited earlier, “When you got doctors and nurses and whoever who says, ‘Well, God works through us,’ that’s who you want on your medical team” (00:09:32), is an example of a belief in providential healing.

#### Prayer As a Gift of Healing and a Gift of Caring

In the Christian Community Model, prayer can be both an instrument of healing and a unit of social exchange. Prayer is seen as having the power to tangibly benefit another; but even when it does not do that, praying for another is understood as an expression of caring and a manifestation of social relationships.

Several participants described feeling tangible effects of prayer on their physical state. According to Wayne, praying during his radiation treatment gave him a “calming feeling” in his body, which had the direct effect of allowing him to endure the extreme discomfort of the position he had to be in.

And after the second day [of treatment], I probably felt there’s no way I’m going to be able to do this by myself. So, I just started praying…And when I did that, it was like the most calming feeling that I’ve ever felt went throughout my body. And from that point on I could start taking the treatments. And when I would go in, and it was like 15–20 minutes at a time, I would pray the whole time I was in there and it helped me get through it (Wayne 00:14:11).

Jane explained that prayer was responsible for recent good news that Jake’s cancer had not spread to his bone marrow:

Not one sign [of bone marrow cancer]. Oh my goodness! And what had happened was…the whole church did a prayer circle for Jake…. He is, every kind of prayer list, you wouldn’t believe, prayer lists that he’s on (Jane 01:07:39).

Aside from its power to heal, prayer is also seen as a unit of social exchange in the Christian Community Model. It is considered a gift given as an expression of caring. Bobby pulled back the curtain on this social fact with an only slightly schismatic slip of the tongue. It is worth noting that Bobby was only recently “saved” after “sixty years of living like hell” (00:39:05), in his words. His narrative opened like many others we collected; with a quantification of the prayers he was receiving. This pattern already points to the exchange-unit nature of prayer. His narrative also concluded in a familiar way, describing the benefits he “has said” he received from prayer. But when the interviewer prompted him to admit to the bodily experience of being prayed for, he balked. After a long pause, he allowed that he had only “told” those who prayed for him that their prayers helped keep him going.

Bobby: By the time I finished everything last year, eight different churches had me on their prayer list…I said so, that’s what keeps you going. I’ve told people, I’ve said, ‘It ain’t necessarily the medical treatment,’ I said, ‘It’s them prayers and good thoughts that keeps you going.’

Interviewer: Right. So, you felt those?

Bobby: [pause] Well, that’s what I’ve told them (Bobby 00:33:48).

Bobby’s insight is that the gift of prayer, like any gift, is not to be snubbed—even if it’s not quite what you hoped for. If prayer is a unit of social exchange, then it is also a form of social capital—the more you have, the higher your social status. In this framework, the quantification of prayers received may be understood as a way of indexing belonging to a Christian Community. The quantification of prayers was very common in the interviews (also see Jane, above). Some interviewees counted prayers in vague terms:

I had cards from so many people. I hung my cards up on my shelves and on days that I felt really, really bad, I would look at those cards and think about the people that was praying for me and thinking about me. That was lifting my spirits (Mary 00:59:21).

Some counted the number of churches praying:

So, you know, I was on a prayer list at a large majority of the churches throughout this state. And I’m very quick to tell anybody that I’m here today because of the power of prayer (Wayne 00:15:56).

And some counted the variety of locations and faith traditions sending prayers:

I have friends in Lexington and friends in Richmond. They would say, I’ll put you on a prayer list, so I had Baptist and Pentecostal and Catholics and Reform and some Mormons. I don’t care who prays for me, I’ll accept all and any prayers (Hazel 00:58:14).

Participants valued prayer, then, both for what it could do and for how it made them feel. Feeling cared for, valued, and as belonging to a community were important ways these patients, survivors, and caregivers coped with illness.

#### Faith and the Moral Imperative of Wellbeing

Within the Christian Community Model, individuals have a moral obligation to protect their own wellbeing, so as not to cause distress for others, and to be prepared to help others. Faith provides one avenue for doing this. Several participants described the restoration or strengthening of their faith as a result of their experience with cancer. For Hazel, several events during her cancer journey helped restore her faith, which, in turn, helped her manage emotional issues that “tormented” her:

So yeah, I was overjoyed, the cancer was not only just contained to the uterus, which was my prayer. It was contained to the endometrial lining...He showed me who He was, and He restored my faith, and He helped me to quit questioning all the why’s that I can’t control in my life, that has tormented me for my whole life (Hazel 00:22:03).

For Wayne, his illness was a cathartic experience that had fundamentally changed his outlook on life, and his behaviors. He claimed that his friends saw the change in him when he became more “tender-hearted” after his illness. Furthermore, he framed his positive attitude and behavior change as not just a personal achievement, but also as a message to share with others. Wayne had been involved with outreach to other cancer patients through the cancer center where he was treated.

You know, I try to look at it as the glass being half full instead of half empty. And when I realize the more I take that approach, to every day in life, the happier it is. And I don’t know that I could have looked at things that way before. As I said earlier, I kind of viewed life as an entitlement…Best thing that you can do is accept it and move on because happiness is a choice…And I found was, is the more people that found out about my situation, the more calls I would receive from people that say, ‘Hey I have a relative that was just diagnosed with cancer. We heard what a positive attitude that you had throughout it, would you mind calling them and just telling them your story?’ (Wayne 00:18:48).

The themes of healing, prayer, and faith were among the most ubiquitous in the interviews, and clearly some of the most important topics to the participants themselves. In Appalachian Kentucky, prayer was described as both instrumental and a unit of social exchange; and faith was viewed as one way for individuals to fulfill the moral imperative of wellbeing.

### FINANCES: FAIRNESS AND VIRTUE

As discussed above, many Appalachians see independence as directly connected to ideas of work and providing for family. A close textual analysis of our interviews suggests that, for our interviewees, the American health insurance landscape poses a challenge for this value system because of its perceived nonlinear mapping between effort and reward: those who work hard are seen as reaping fewer health benefits than those who work little. Thus, through the lens of the Independence-Through-Work Model, interviewees saw the financial problems associated with their illness as unwarranted, unfair, and illogical.

In the Independence-Through-Work Model, the contrast between independence and dependence is not just a matter of fairness, but also a matter of personal virtue. People who value Independence-Through-Work are seen as good, while those who do not are seen as bad. The value system governing health insurance benefits in America was seen by many interviewees as requiring immoral behavior—such as lying or laziness—to function.

The stories below illustrate the sometimes-uncomfortable fit between the Appalachian worldview and the worldview that interviewees believe undergirds the structure of the American healthcare benefits and insurance industries, and how cancer patients navigated that difficult fit in times of financial need.

#### Perceptions of Threat to the Independence-Through-Work Model

Patients described an inability to exercise their values related to Independence-Through-Work in the context of the healthcare benefits system. Even though they felt they had kept up the end of the deal over which they had control—working—they found themselves trapped within a different system they saw as not rewarding this hard work. As Wanda and others claimed, the healthcare benefits system seemed to punish their hard work and reward “laziness,” instead. This other system was also perceived as punishing the “virtuous” act of marriage, rewarding instead divorce and cohabitation, deemed as not “virtuous.” In the excerpt below, Wanda used the metaphor of “falling through the cracks” to locate herself and her sick husband as trying to operate within the unfamiliar system and failing. She then pinpointed their history of hard work and marriage as the reasons she believed they were unable to get financial assistance for his care.

We were unable to get any kind of financial assistance help, something to help us with our out-of-pocket copays. We had no other income other than mine, and he had to sign up on disability. We fell through the cracks because where me and him was married, and we both had income coming in, they don’t even take in consideration all your finances that you have… I had a list of all these places I would contact to see if we qualified for any kind of help, but where [i.e. because] we worked, we couldn’t get anything (Wanda 00:57:50).

According to Mary, work and access to health insurance are mutually exclusive in the current system. She cited one previous version of the system, “Obama Care,” where she believed that work was valued. From the perspective of the Independence-Through-Work Model, the mutual exclusivity of work and benefits is contradictory.

And you can’t qualify for the medical cards if you’re working, so it’s really a hard situation. It’s the reason I hated to see what’s happening at, you know when we had the, what they coin the Obama Care [Affordable Care Act], at least you could continue to work and get a decent insurance. Now that’s starting to slowly trickle away again (Mary 00:33:01).

Another feature of the health benefits system, according to interviewees, is its unreliability. For Mary, for example, this caused fear. She saw the system as unreliable both because it threatened to abandon her just in her time of need, and because it could not be trusted to keep its promise of no penalties for “pre-existing problems.”

Even though I felt lousy and my health was deteriorating, I had to continue to work and I worked part-time for the last two years to get my health insurance basically. If I had quit my job due to my health, my husband works at the college, we would not have qualified for any health insurance. Except if he would have picked me up, which once you have a cancer diagnosis, even though they say there’s no pre-existing problems, there is pre-existing problems. It was scary for me (Mary 00:44:11).

#### Perceptions of Threat to the Christian Community Model

Besides challenging the Independence-Through-Work Model, interviewees attributed certain values to the national healthcare benefits system, values which appear to violate key assumptions of the Christian Community Model. Whereas the Christian Community Model values Christian social relations, the health benefits system was perceived as valuing transactional, opportunistic social relations. As Wanda described, “the system” appears to allow and even reward what she saw as dishonesty and the disrespectful misrepresentation of social relationships.

If me and him had divorced when all this come down, which we wouldn’t have done it anyway, but people know how to do things. You divorce and then you let the patient sign up for all this free stuff, and they get all this free stuff. It ain’t my faith to do that, you know? That’s the system we live in today, the society and system that’s in this country today (Wanda 01:05:13).

Similarly, Jane recounted with incredulity a conversation she had when trying to obtain social security for her son. Her story implied moral outrage at the understanding that lying might have resulted in more money for her son, and at the system’s apparent blindness to the social inappropriateness of suggesting she charge her sick son for her help.

We had to fight for disability. It’s frustrating because you want to do everything you can for your loved one. So, our philosophy is we always tell everybody the truth. We don’t want a dime that we don’t deserve….The lady asked me, ‘Do you charge him rent?’ ‘No, he hasn’t worked in four months.’ ‘Well, are you going to make him pay you back for the rent?’ ‘No, my children don’t pay me rent.’ We all found out later, if I had said, ‘Yes,’ he would have gotten $250 more per month. They gave him less Social Security because I don’t charge my son, my cancer patient son (Jane 00:17:48).

Financial difficulties were among the most frustrating for the interviewees dealing with cancer because they felt forced to interface with what they saw as an irrational, immoral, but powerful system in which they felt stripped of agency. Furthermore, they perceived the system as undergirded by a worldview that contradicts, and appears to even threaten, key values in both the Independence-Through-Work and Christian Community Models.

### KNOWLEDGE AND POWER IN HISTORICAL CONTEXT IN APPALACHIA

In understanding any worldview, one of the most fundamental themes is the creation and sharing of knowledge. The theme of knowledge is entwined with the theme of power. What counts as valid knowledge? Who has the power to create and disseminate knowledge? In eastern Kentucky, the Appalachian Modern Worldview provides a framework for answering these questions, but a framework that is hybrid and complex.

In the healthcare context of eastern Kentucky, the legitimacy of different kinds of knowledge and power is contested. Respect for scientific knowledge and the authority of “experts” coexists with a healthy skepticism of anonymous sources of authority and a respect for the individual’s ability to reason for himself or herself. An expectation that individuals exercise agency in obtaining knowledge coexists with Christian ideals of surrendering agency to God. An understanding of the empowering possibilities of scientific knowledge coexists with a recognition of how such knowledge has been used historically to marginalize the people of Appalachia.

In discussing their approaches to accessing information and communicating with medical professionals, participants invoked a fraught history of struggles for knowledge and power in Appalachian Kentucky. The stories that follow underscore a deep cultural tension between the desire for (individual and regional) self-determination and the need for outside expertise.

#### Participants’ Varied Approaches to Information Gathering

Box 2 **Words of Wisdom**

For Wayne, the combination of online resources and a trusted medical professional who was also a friend helped him make vital decisions about whether to undergo surgery. And [my oral surgeon] said: “Over the next several weeks, you’re going to have doctors telling you what they can and they cannot do for you. What you need to remember is, at the end of each night, you’re going to have to live with the consequences of whatever treatment you have done.” And he gave me some websites to look at. And that was probably the most beneficial advice that I got throughout the whole process. (Wayne 00:04:06)

At one end of the spectrum, some patients explicitly rejected scientific knowledge as an expression of faith. Wanda’s husband, for example, did not want to know his own prognosis. She described this request in terms of his prioritization of spiritual ways of knowing over scientific ways of knowing.

We actually, my husband never wanted to know his prognosis because he always had faith in God and he always believed that he was going to get through this. I mean, he really did. I really did too. We had our faith during this, so he didn’t want to know no prognosis…Up until the end, he never knew his prognosis (Wanda 00:43:26).

Bobby’s attitude toward knowledge reflected traditional Appalachian independence, while also embracing modern, scientific ways of knowing. In Bobby’s characterization, individuals have the responsibility, ability, and agency to educate themselves. He positioned himself as both “old school” and modern, saying, “Information is very helpful.” Bobby rejected the approach of people like Wanda’s husband, even pointing out that knowledge can have medical benefits.

Well, people can educate themselves. They shouldn’t just gobble what they’re told...If I don’t know something, I’ll ask questions, or I’ll research, or I’ll grab a book. I’m old school, I like that piece of paper in my hand, I like that magazine in my hand, I like that book… Information is very helpful. I know there’s times you go to the doctor, you don’t want to know something. But I’ve been told sometimes it’s best to know something, to catch something early (Bobby 00:48:57).

In contrast to Bobby’s agentive perspective, some patients saw it as the doctor’s responsibility to educate them. This perspective aligns with an Appalachian Modern notion of hierarchical social relations, in which authority figures are the arbiters of knowledge. Harry all but blamed his doctors for his cancer because they failed to force him to get a colonoscopy:

Well, the blame lays with me for not having—but the doctors truly have—with the age and everything else they should have insisted. Not just asked that. ‘Harry, I really want you to have one [a colonoscopy]’ (Harry 00:37:36).

Of course, at the other end of the spectrum, patients with a more classically modern worldview perceived the doctors-as-authority-figures trope as patronizing. Mary explained that negative stereotypes of Appalachians cause some outside doctors to think of them as children. In her characterization, the stereotype of the “childlike Appalachian” is rooted in their perceived lack of knowledge.

And that’s, they pick up stories like this from eastern Kentucky and then that’s how we’re portrayed, and I think that sometimes we have doctors that come in from the other areas for eastern Kentucky and they love it here. They love staying. Once they get to know the people, they love the people. But they come in with the idea that, ‘I’m going to have to take care of these kids, they don’t know nothing’ (Mary 00:36:53).

This excerpt from Mary also points out another interesting pattern. Many of the doctors in Appalachian Kentucky are foreign-trained. A special program allows foreign-trained doctors to serve for 2 years in Appalachia in order to qualify for an expedited permanent residency process. One doctor, who claimed to be the “only U.S.–trained specialist” in the chain of hospitals for which he worked, explained that although many doctors stay in eastern Kentucky for the 2-year program and then leave, a sizable number have made permanent homes in the area and “people are used to them.” Even so, there can be communication challenges between patients and some newly-arrived doctors. This doctor explained that patients often told him they preferred him because they had difficulty understanding the other doctors. The abundance of foreign-trained doctors in eastern Kentucky hospitals also adds an inter-cultural communication dimension to some of the medical domain-related challenges patients may face when communicating with doctors.

Mary was one of the strongest proponents of the idea of the intellectual parity of patients and doctors. This value was rooted in her personal experience having been both an educated cancer patient, and an educator of patients in her job in a healthcare profession. In addition to expecting doctors to “give patients the benefit of the doubt,” she criticized the ways in which doctors’ power was inflated by the healthcare system through the uneven distribution of information.

It’s interesting, [my general practitioner] got a different reading or a different result than I received in the mail...If I had received that in the mail, I’m educated enough to know I may need to probably have something checked. That’s another thing that I feel that our radiology departments just don’t give the patient the benefit of the doubt that they will follow through and take care of themselves. But thankfully my doctor decided to do that instead of waiting another six months (Mary 00:15:17).

Patients characterized doctors as being potentially defensive in the face of challenges to their control over medical information and authority. Describing his own agentive information-seeking, Bobby likened his ability to read lab results to a doctor’s. He subtly conveyed that providers might be expected to feel threatened when their monopoly over expertise is challenged by patients like himself—although the particular providers in this story denied being offended. In this narrative sleight of hand, Bobby drew out a cultural tension while maintaining social harmony among his characters.

I thought well, I’ve talked to different nurses, they always are amazed how I can read my lab work like a doctor. I want to know what a CT scan result is. The doctors and nurses has told me, they said, ‘Bobby that’s great. We want patients to be informed and ask questions.’ They said, ‘You don’t offend us’ (Bobby 00:03:03).

Similarly, Jane suggested that doctors can be expected to defend their monopoly over expertise —all while complimenting her son’s doctors for not behaving in this way. Jane was the only interviewee to explicitly reference the relationship between knowledge and power.

The whole team is like sitting down with your best friend, and they answer every question...And what’s awesome is nobody talks above our heads. They don’t make us feel stupid…And I liked that they empower us. They gave us...I don’t know if you’ve seen them, American Cancer Society gives out an organizational folder? (Jane 00:26:33).

For Jane and her son, empowerment came not just from the knowledge itself, but from how it was distributed: in a way that made her feel like she was talking with friends. These examples also illustrate, however, the ongoing nature of the historical struggle over knowledge and power in Appalachia, in which it cannot be taken for granted that all experiences will be so positive.

Box 3 **Words of Wisdom**

But I’m really fortunate to have the doctor I’ve had, and I’ve had him for over twenty years and he always listens. He takes my word for a lot of things because he knows I try to stay educated about my health and I streamline my medicine through him now, so I don’t have all these different specialists prescribing different medicines. He prescribes all of it. I always try to educate: When I worked at the health department, I educated people on that, that you take control of your health, you keep your records and they belong to you. You can get copies, and then as far as your medication, make sure one doctor’s prescribing all your medication so you’re not having one doctor giving you something that counteracts something else that somebody else is giving you. It’s just a matter of taking care of your health (Mary 00:42:08).

#### Preservation Versus Progress in Development Projects

The themes of knowledge and power are also particularly relevant for initiatives that seek to innovate in Appalachian Kentucky. Such initiatives should be cognizant of the historical conditions that have given rise to negative attitudes toward outside expertise and seek ways to decouple the relationship between power and various kinds of knowledge.

In the Appalachian Modern Worldview, there is a resistance to modernization inasmuch as it is perceived to threaten key regional values. At the same time, there is recognition that development projects could restore economic independence to Appalachia. This tension between preservation and progress characterizes the problem of how to address several barriers to improving cancer care in eastern Kentucky, including transportation barriers, broadband access and adoption gaps, infrastructure development, and economic development.

#### Transportation in a Sacred Landscape

Transportation was cited as the number one challenge to providing cancer care to rural patients by almost every community partner we talked to. Beverly, the director of a nonprofit organization dedicated to helping cancer patients access resources, spoke extensively of the practical problems of providing transport to patients. Her organization had experimented with gas cards, home visits, and even transporting patients themselves. But each strategy ran up against bureaucratic red tape. As she investigates the potential of a partnership with a ride-share company in the future, she reports that transportation is the number one problem her clients face.

**Figure 2:**
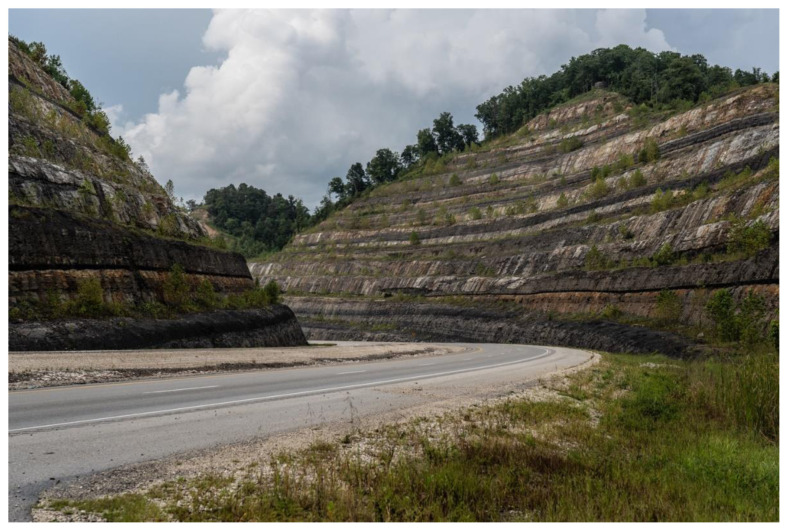
In eastern Kentucky, even basic infrastructure requires colossal feats of engineering. Modernization projects alter the landscape in unmistakable ways, leaving visual evidence of the tension between preservation and progress. Photo credit: Melanie McComsey.

However, the modern highways that improve safety and access to resources also erode the seclusion valued by many eastern Kentuckians (Figure 2). In the vernacular of Modernity, rurality is often portrayed by some as a “burden” that needs to be “compensated for.”^23^ But this perspective contradicts traditional Appalachian values because it rejects the possibility of a place that could be both rural and modern. Appalachian Modernity allows for an alternative perspective in which rural life is seen as having intrinsic value, which should be enhanced through, or preserved despite, modernization.

Indeed, the landscape of eastern Kentucky is a powerful symbol of traditional Appalachian values, and we heard interviewees talk about it in reverential terms. Its ruggedness points to the idealized characterization of the Appalachian native—resourceful, strong, and independent. Its impenetrability points to a regional success story of resisting outside influence—of self-sufficiency and the right to self-determination. And the beauty of the landscape continues to symbolize a sort of alternatively-modern animism that co-exists with other religious and secular frameworks.

Reflecting on her cancer experience, Penny mused about her love of the mountains. She outlined the direct relationship between the strength of the mountains and human strength and expressed a belief in their healing powers.

I have become awakened to what I have missed in my life. I should have done things when I had the energy and the wellness to do them. Now it’s almost, ‘I’m not going to say it’s too late, it’s just going to be more of a challenge to get it done...Taken trips, going to the beach. Of course, there is nothing like the mountains. I love the mountains…The strength comes from the mountains. They were given so many years ago and they are still standing strong. It says in the Bible, the strength comes from the mountains. I truly believe that it is a good healing place (Penny 09:24).

**Figure 3:**
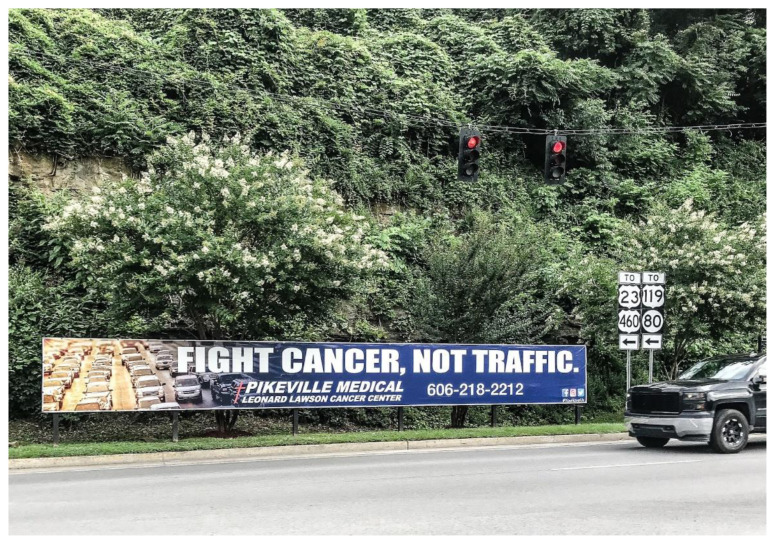
The fear of urban traffic among eastern Kentuckians is so ubiquitous that it was leveraged in an advertising campaign for Pikeville Medical Center, which urged the public to “fight cancer, not traffic” by staying local. Photo credit: Melanie McComsey.

Interviewees alluded to their ambivalence toward modern transportation scenarios through a recurring trope of traffic “battle narratives.” For many eastern Kentuckians, their fear of driving in “traffic” in the urban Lexington environment is both palpable and worn as a kind of badge of honor of local authenticity. Community partners told us that patients often brought up traffic as a primary barrier for them to travel to Lexington, and several interviewees told their own traffic battle narratives. For example, Jane claimed that her daughter cried the first time she drove in Lexington. The fear (or narrative of fear) of urban traffic among eastern Kentuckians is so ubiquitous that it was leveraged in an advertising campaign for Pikeville Medical Center, which urged the public to stay local and “Fight Cancer, Not Traffic” (Figure 3).

Although it may be impossible for Appalachians to preserve their seclusion, privacy, independence, and authenticity while also addressing transportation barriers, the paradox is something that must be grappled with in development projects that seek to improve access to urban resources through transportation infrastructure.

#### The “Poverty is Elsewhere” Narrative As a Strategy of Resistance

Median household income is more than $20,000 lower in Appalachian Kentucky ($33,492) compared to national estimates ($53,889). Appalachian Kentucky has higher percentages of children (34%) and adults (25%) living in poverty compared to national data (22% and 15%, respectively), making it among the most economically distressed regions in the country.^24^ In 2017–2018, 69% of Appalachian Kentucky counties were classified as “distressed,” with economic indicators in the lowest 10% of all U.S. counties. Only 14% of Appalachian Kentucky adults age 25 and older hold a bachelor’s degree or higher compared to 30% nationally.^25^

Dire-sounding statistics such as these about poverty and other kinds of “deficiencies” are often relied upon for distributing resources in development and modernization projects, yet Appalachians resist such depictions of themselves as impoverished. One of the most ubiquitous narratives heard from both interviewees and community partners was that “poverty is elsewhere,” a narrative that seeks to deny poverty for oneself and displace it elsewhere. This concerned both individual poverty and community-level poverty. No one we spoke with would characterize himself or herself as being “poor” or as having financial troubles outside the realm of the health benefits system. This narrative is complex because it is rooted both in regional solidarity against outsiders’ “hillbilly” stereotypes, and in regional factionalism along class lines.

Penny exemplified both of these stances in a conversation with the interviewer about her hometown. When the interviewer claimed to have visited an area near Penny’s home, Penny distanced herself from people the interviewer may have met. She defended herself against a certain representation of people from her area. Penny positioned herself as different from “trashy” people, framing their failing as a moral one that aligns with the Independence-Through-Work Model: they don’t “seem to want to better themselves.” For Penny and other Appalachians, then, local class distinctions have less to do with measurable wealth and poverty, but with industriousness and self-presentation.

Interviewer: I’ve been [to your county]. I actually, I had a nice lady who took me on a tour of that county and took me up Baker Holler.

Penny: Oh my gosh! That does not represent all of us. I’m sorry to say, or I’m glad to say.

Interviewer: Okay, how not?

Penny: Just, unkept houses, trash, just people that doesn’t seem to want to better themselves…Because of the cleanness, you know, it’s not a disgrace to be poor but, it is to be trashy. You can always clean and mop and take garbage off but, to just let it pile up, it’s just sad (Penny 00:41).

For the reasons outlined above, it appears that to admit to being poor is to reject Appalachian morality and personhood. Typical statistical measures of poverty do not align with local measures of effort. This cultural logic as applied to people was also applied to entire communities and counties. I came to think of one interesting phenomenon as “the race to not be worst.” In each county I visited, residents described neighboring counties as “even poorer.” In Johnson County, I heard that Martin County is “the poorest in Kentucky.” In Martin County, I heard that Clark is “the poorest county in the nation.” In Clark County, I was told I “wouldn’t believe” the state of things in Laurel County. Regardless of the accuracy of these statements, residents consistently denied poverty as a feature of their own communities, while displacing it on others.

The “poverty is elsewhere” narrative is apparently used by Appalachians to refuse resources, resisting would-be developers, and protecting Appalachian values and resources. Aside from diverting outside agents of development projects, I heard several stories from local community partners about their inability to distribute existing resources even in their own communities. One community partner told a story about a program to distribute cash cards for prescription medication, but people would not accept the cards. Another community partner told a similar story about her participation in a program to distribute shoes to school children in her home county in eastern Kentucky. Voicing her own neighbors, she said she heard the same refrain over and over, “Aw, honey, you give those to someone who really needs them.” In the local vernacular, this attitude is often described as “pride.” Interpreted through the lens of Appalachian cultural models, the denial of poverty is a collective strategy to live by the values of the Independence-Through-Work Model, to maintain the distinctly Appalachian spirit of modernization, and to maintain local control over the way resources are distributed.

## CONCLUSIONS

One strategy for utilizing ethnographic insights in healthcare innovation projects is to frame processes of innovation and implementation in terms of local values. These values can guide choice of design elements for solutions or for implementation plans. This method may be used both to generate ideas and as a system of checks and balances for solution prototypes. As solutions are developed to be piloted in the field, they may be analyzed for coherence within the key cultural frameworks outlined in this report.

As an example of such an approach in the context of cancer distress monitoring, patient–provider communication about “distress,” including in the use of distress monitoring tools, could align with Appalachian understandings of suffering or more effectively translate between Appalachian and clinical understandings of distress, such as “nerves” versus “anxiety.” Furthermore, the insight that a significant source of patients’ financial frustration comes from their encounter with the health insurance industry, which they perceive as “illogical” and “immoral,” suggests an opportunity to facilitate a form of intercultural or intersystemic translation when innovating in healthcare spaces in Appalachia.

The unique transportation challenges that were identified, including the “fear” of urban traffic, also suggests a need to better leverage broadband and other technology to offer on-demand care to Appalachians in their homes, churches, and community centers where familial and social support systems already exist and are trusted. More generally, for development projects, insights discussed here about the stigmas attached to receiving certain kinds of “help” in Appalachia present an opportunity to reframe approaches to implementation. Reframing “help” in the context of what is referred to locally as “pride,” by leveraging values of “offering,” work, and reciprocity, could improve adoption and longevity of solutions. This is a particularly powerful insight for more broadly introducing connected health and ensuring sustainability over time, given traditional lags in Internet adoption in rural communities, where consumers report a perceived lack of relevance to their everyday lives as one key barrier to adoption.

Another approach to integrating ethnographic insights in healthcare innovation is to tailor solutions to local patterns of behavior. Designs that leverage people’s existing behaviors or integrate into patterns of behavior with minimal disruptions are more likely to be adopted and used. The findings presented here suggest ways to integrate future design solutions into existing behaviors common among Appalachian cancer patients in order to improve functionality.

For example, the insight that cancer patients rely not only on immediate family but on extended “church families” and “work families” to help with practical aspects of their care suggests an opportunity to explore solutions that facilitate communication, including technology-driven and in-person connections among patients, caregivers, and other trusted intermediaries. Individual-centric solutions to symptom management may be less successful in Appalachia than solutions that target extended communities of caregiving. Connected health solutions may be a force multiplier in this regard, responding to the need among some of those interviewed for more detailed health information, education and community support as their condition unfolds. The insights that patients may use prayer for self-comfort during treatment, and that receiving prayers from others helps alleviate some emotional suffering, suggest an opportunity to explore the role of cognitive–behavioral approaches to managing some forms of distress.

The insight that there appears to be considerable variation in information-seeking and communication behaviors suggests several opportunities. First, there may be an opportunity for doctors to be able to tailor methods of information-sharing and communication to patient practices and expectations. Second, behaviors that are most “empowering” for patients, such as having access to their own records, may be amplified and used as starting places for the innovation process. Finally, the extreme variation in behavior and worldview in this realm suggests an opportunity for effecting behavioral change, if needed. Cultural domains in which extreme within-culture variation is observed are often most amenable to change because alternative models have been generated inside the culture.

Finally, and perhaps most importantly, ethnographic insights may guide how the innovation process itself is theorized and implemented. Insights about the continuing relevance of the history of exploitation of Appalachians on their own land points to an opportunity to reimagine what a development initiative can be. Some Appalachians are resistant to outside institutions and development projects that seem to threaten traditional values. Approaching community members as collaborators rather than research subjects may begin to break down some of the implied power differentials in multi-institutional projects. Approaching modernization and development in a way that amplifies and complements local values can help build relationships in communities. Awareness of issues related to trust, power and community empowerment are also key to project sustainability.

In this vein, coalition-building is a vital, ongoing component for healthcare innovation projects. The research conducted as part of this ethnographic work, for example, is intended as a preliminary step of partnership formation in a community-based participatory research process. Subsequent steps in the coalition building process include: the formation of the partnership, identification of opportunities, assessment of community strengths, sharing findings, seeking feedback on findings, and implementation.^26^

Also, in this vein, there is an opportunity for outside projects to act in a supporting role for local, grassroots innovations. Innovation projects of this kind, spurred and led by real patients, may already be taking place in Appalachian communities. Users whose current needs forecast future general needs have been called “lead users.”^27^ Lead users in healthcare and technology settings are patients, caregivers or providers at the forefront of processes, who can serve as early adopters and identify gaps in design.

For example, Mary, one of our interviewees and a breast cancer survivor, also had a long career in healthcare. Her professional experience and contacts made it possible for her to create a solution to one of the problems she faced during her illness: her cancer was missed for several years because she had been getting analog mammograms. As she explained:

I worked with the Breast Center and just implored them to come to this area and offer the mobile unit for our ladies because we—I knew that there was probably a whole lot more people out there because we had a very high rate of breast cancer in this area. They have been coming to the health department once a month and doing them, with the mobile unit and doing the imaging that’s more than just the analog (Mary 00:13:29).

Mary also arranged a partnership with the health department in a neighboring county so the mobile unit would be sure to have a full roster when it arrived in Kentucky. And she secured funding from a nonprofit organization to cover the extra costs of a mobile mammogram. One difficulty she faced was overcoming funding obstacles to bringing a mobile unit from Kentucky; ultimately, she had to seek a mobile unit from a neighboring state. She recognized the unique contribution she was able to make:

But I don’t know if that would have ever taken place if it hadn’t been for me and a couple of other ladies who had breast cancer to work with them and try to get them to come (Mary 00:30:12).

Mary’s story points to opportunities to seek out examples of such “lead users,” perhaps among people in healthcare and technology fields. Grassroots, or lead-user, innovators may inspire solutions as well as potentially take leadership roles in community-powered innovation. It is important for development projects to also recognize the unique contributions of local innovators, and the unique culture that shapes how Appalachians imagine their own futures.

This paper represents an initial foray into and a model for innovation projects that seek to break the mold of the typical development encounter paradigm. As a multi-institutional collaborative representing a panoply of classically modern viewpoints and motivations, L.A.U.N.C.H. is well positioned to innovate not only at the intersection of healthcare and connectivity, but also in the space of community-driven, collaborative design. Ethnographic projects, as presented in this paper, serve to foster mutual understanding, the alignment of perspectives and goals across stakeholders, and local coalition-building; these are but the first steps in an innovation process that will include collaborative co-design and implementation phases. And while this demonstration project, to improve connected cancer care for rural Kentuckians, may seem specialized, we see it as no less than a model for how every community may come to practice the design of itself, to reimagine and reconstruct local worlds.^28^

## Figures and Tables

**Table 1:** Ethnographic Interviews

Interviewee	Role	Gender	Patient’s Age at Diagnosis	Patient’s Cancer Type
Bobby	Patient	M	60	Pancreatic
Claire	Caregiver	F	50s, 60s	Bladder (mother); Lung, brain (father)
Holly	Caregiver for husband, Harry	F	See Harry	See Harry
Hazel	Survivor	F	54	Uterine
Harry	Patient	M	67	Colon
Jane	Caregiver for son	F	20	Rhabdomyo-sarcoma
Laura	Survivor	F	36	Breast
Mary	Survivor	F	40s	Breast
Penny	Patient	F	50s	Sarcoma bicep, lung
Wanda	Caregiver for husband	F		Lung, brain
Wayne	Survivor	M	52	Throat

## References

[1] NIH and CDC. National Cancer Institute and Centers for Disease Control and Prevention 2020 State Cancer Profiles 2012–2016 www.statecancerprofiles.cancer.gov

[2] ARC Health Disparities in Appalachia 2017 https://www.arc.gov/research/researchreportdetails.asp?REPORT_ID=138 10.1377/hlthaff.2017.1243 29200341

[3] FCC Broadband Deployment Report Federal Communications Commission 2018 https://www.fcc.gov/reports-research/reports/broadband-progress-reports/2018-broadband-deployment-report

[4] Siegel RL Jemal A Wender RC Gansler T Ma J Brawley OW An assessment of progress in cancer control CA: a cancer journal for clinicians 2018 68 5 329 39 30191964 10.3322/caac.21460

[5] Hesse BW Ahern DK Ellison M Barn-raising on the digital frontier: The L.A.U.N.C.H. Collaborative J Appalach Health 2020 2 1 6 20 DOI: 10.13023/jah.0201.02. PMC9138843 35769536

[6] President’s Cancer Panel Improving Cancer-Related Outcomes with Connected Health: A Report to the President of the United States from the President’s Cancer Panel Bethesda MD 2016

[7] Kleinman A The Illness Narratives: Suffering, Healing and the Human Condition Basic Books 1988 10.1097/ACM.0000000000001864 28952997

[8] Geertz C The Interpretation of Cultures New York Basic Books 1973

[9] Escobar A Encountering Development: The Making and Unmaking of the Third World Princeton Princeton UP 1995

[10] Eller RD Uneven Ground: Appalachia Since 1945 Lexington University Press of Kentucky 2008

[11] Durkheim E The Rules of Sociological Method Lukes Steven Halls WD New York The Free Press 1982

[12] Topos Partnership. Independence, pragmatism, and the flow of money: Findings from ethnography in central Appalachia www.topospartnership.com 2015

[13] Hall S Formations of Modernity Polity Press The Open University 1992

[14] Knauft BM Critically modern: Alternatives, alterities, anthropologies Indiana University Press 2002

[15] Becker JS Selling tradition: Appalachia and the construction of an American folk, 1930–1940 Univ of North Carolina Press 1998

[16] Hatch E Modernity with a mountain inflection J Appalachian Studies 2008 14 1/2 145 59 https://www.jstor.org/stable/41446806

[17] Keefe SE Theorizing modernity in Appalachia J Appalachian Studies 2008 14 1/2 160 73 https://www.jstor.org/stable/41446807

[18] Peine EK Schafft KA Moonshine, mountaineers, and modernity: Distilling cultural history in the southern Appalachian mountains J Appalachian Studies 2012 18 1/2 93 112 https://www.jstor.org/stable/23337709

[19] Lewis RL Billings DB Appalachian culture and economic development: A retrospective view on the theory and literature J Appalachian Studies 1997 3 1 3 42

[20] Basch E Deal AM Dueck AC Overall survival results of a trial assessing patient-reported outcomes for symptom monitoring during routine cancer treatment JAMA 2017 318 2 197 8 10.1001/jama.2017.7156 28586821 PMC5817466

[21] Gustafson DH DuBenske LL Namkoong K An eHealth system supporting palliative care for patients with non–small cell lung cancer: a randomized trial Cancer 2013 119 9 1744 51 23355273 10.1002/cncr.27939 PMC3684251

[22] Commission on Cancer Cancer Program Standards: Ensuring Patient-Centered Care, 2016 Edition https://www.facs.org/qualityprograms/cancer/coc/standards

[23] Salemink K Strijker D Bosworth G Rural development in the digital age: A systematic literature review on unequal ICT availability, adoption, and use in rural areas Journal of Rural Studies 2017 54 360 71 10.1016/j.jrurstud.2015.09.001

[24] US Census 2016 American Community Survey (ACS): 2011–2015 ACS 5-year Estimates https://www.census.gov/programs-surveys/acs/technicaldocumentation/table-and-geography-changes/2015/5-year.html

[25] Appalachian Regional Commission County Economic Status and Number of Distressed Areas in Appalachian Kentucky, Fiscal Year 2018 https://www.arc.gov/images/appregion/economic_statusFY2018/CountyEconomicStatusandDistressAreasFY2018Kentucky.pdf

[26] Butterfoss FD Coalitions and partnerships in community health John Wiley & Sons 2007

[27] Von Hippel E Lead Users: A Source of Novel Product Concepts Management Science 1986 32 7 791 805

[28] Escobar A Designs for the Pluriverse: Radical Interdependence, Autonomy, and the Making of Worlds Durham Duke UP 2017

